# Prototyping a Chatbot for Site Managers Using Building Information Modeling (BIM) and Natural Language Understanding (NLU) Techniques

**DOI:** 10.3390/s23062942

**Published:** 2023-03-08

**Authors:** Will Y. Lin

**Affiliations:** Department of Civil Engineering, Feng Chia University, Taichung 407, Taiwan; weiylin@mail.fcu.edu.tw; Tel.: +886-973-531-289

**Keywords:** chatbot, Building Information Modeling (BIM), Natural Language Understanding (NLU)

## Abstract

Amidst the domestic labor shortage and worldwide pandemic in recent years, there has been an urgent need for a digital means that allows construction site workers, particularly site managers, to obtain information more efficiently in support of their daily managerial tasks. For workers who move around the site, traditional software applications that rely on a form-based interface and require multiple finger movements such as key hits and clicks can be inconvenient and reduce their willingness to use such applications. Conversational AI, also known as a chatbot, can improve the ease of use and usability of a system by providing an intuitive interface for user input. This study presents a demonstrative Natural Language Understanding (NLU) model and prototypes an AI-based chatbot for site managers to inquire about building component dimensions during their daily routines. Building Information Modeling (BIM) techniques are also applied to implement the answering module of the chatbot. The preliminary testing results show that the chatbot can successfully predict the intents and entities behind the inquiries raised by site managers with satisfactory accuracy for both intent prediction and the answer. These results provide site managers with alternative means to retrieve the information they need.

## 1. Introduction

Due to the domestic labor shortage and worldwide pandemic in recent years, there has been an urgent need for construction site workers, particularly site managers, to efficiently obtain accurate information that supports their daily managerial tasks. The lack of access to required information may lead to poor project coordination, which could significantly affect the overall project performance [1]. In the past few decades, various commercial or customized construction information systems have been developed to serve as important means for users to perform such a search. Most of these systems are based on a form-like user interface because the users can easily understand the idea of the interface [2,3]. For construction site workers who often stay and walk back and forth around the site, however, these form-based interfaces require many finger motions such as key hits and clicks, which can be inconvenient. Therefore, this inconvenience significantly reduces their willingness to use these information systems.

Conversational AI, also known as a chatbot, is a type of software agent designed to emulate human conversation and offer automated assistance to users. Chatbots have gained widespread attention due to the growing demand for efficient and convenient customer service in various industries, such as e-commerce, finance, and healthcare.

Chatbots can be classified into two categories in terms of objectives: task-oriented and non-task-oriented. Task-oriented chatbots are highly compatible with the requirement of information retrieval for construction site workers. Efforts have been invested in research and development to adopt this technology in recent years. Tsai et al. [4] developed a chatbot with a friendly user interface and an intent-matching mechanism to help users retrieve water-related disaster information for decision-making. Another research [5] that aims to improve the efficiency and user experience of facility management also developed a chatbot that integrates NLP, Building Information Modeling (BIM), and ontological techniques to increase the efficiency of information retrieval. Cho et al. [6] proposed a chatbot-assisted construction daily report system, which collects conversations among subcontractors on instant messaging applications and automatically generates daily reports for general contractors. This study adopted Natural Language Understanding (NLU), which can understand the users’ intents for subsequent automatic extraction of the necessary information. Some studies [4,5] have leveraged instant messaging platforms to enhance the ease of use and usability of information systems, but they lacked Natural Language Processing (NLP), which resulted in poor performance. Other studies have adopted self-implemented NLP techniques [5,6] to extract information from textual data sources, but their lack of natural language understanding models results in high demand for manual input to maintain the corpus and limits their systems’ ability and applicability. While a few chatbots have been developed taking advantage of NLU models [6], those that also retrieve geometric data of buildings that are essential for construction site workers are still rare.

If NLU could be used by chatbot developers to automatically identify the intent behind the inquiries of construction site managers during their daily work, then the NLU-based chatbots would take spoken language as input without requiring excessive finger motions and specific keywords, thus increasing the ease of use and usability of the information system and relieving the burden of system implementation. Moreover, when taking advantage of BIM’s capability to represent both the attributes and geometry of buildings in a tabular schema, chatbot developers can easily retrieve building component dimensions, providing construction workers with easy access to essential information needed for their daily tasks on construction sites. The proposed approach may contribute to maintaining or even improving construction performance during periods of labor shortages.

In summary, this study aims to achieve two objectives:

1. To establish a demonstrative NLU model for construction site managers by collecting intents and utterances behind common inquiries about building component dimensions during their daily routines.
2. To prototype a chatbot, named JULIE, that can recognize both the intent and entities behind inquiries with prediction confidences based on the aforementioned NLU model and ultimately retrieve the answer and respond to site managers.

## 2. Literature Review

### 2.1. Related Studies

Conversational AI, also known as a chatbot, is a software agent designed to emulate human conversation and provide automated assistance to users. Chatbots have gained widespread attention due to the growing demand for efficient and convenient customer service in various industries, such as e-commerce, finance, and healthcare. Chatbots can focus on a specific task such as ordering food, booking a flight, or scheduling an appointment, which are called domain-specific or goal-oriented chatbots, also known as task-oriented. Conversely, there are non-task-oriented or open-ended chat systems that are for chit-chat and do not achieve a specific task or objective. Both chatbots are designed to process and understand human language input with the goal of extracting meaning and making predictions based on that meaning, but there are some key differences between them. Non-task-oriented chatbots, also known as virtual assistants, primarily use AI and big data technologies to provide users with a general answer through QA-like conversations between humans and machines. The first and most widely known virtual assistant is Siri launched by Apple in 2010. Consequently, many companies have launched different virtual assistants such as Amazon’s Alexa, Microsoft’s Cortana, Google Assistant, and Samsung’s Bixby. A new generation of non-task-chatbot, ChatGPT [7], can even understand complex questions and generate a complete text as human writers create on a wide range of topics, instead of just simple answers with several sentences. Those non-task-oriented chatbots are trained on a massive dataset that is sourced from the internet and includes a diverse range of text. Because they cover almost all kinds of topics, non-task-oriented chatbots are mostly based on a deep learning model that is pre-trained on a large corpus of text data. On the contrary, task-oriented chatbots only perform a particular task, and therefore, they do not require as massive a training dataset as non-task-oriented chatbots, and most of them use machine learning algorithms or rule-based methods to understand the context. Like those chatbots serving customer service in the above-mentioned industries, the chatbot developed by this research is also task-oriented and serves in the construction industry. Thus, chatbots in the following text are referred to as task-oriented.

Although previous studies [8,9] have reported that most users agree that chatbots help increase the usability of software applications and have been implemented in many fields, their use in the construction industry has just started to appear in recent years as a way to improve communication, collaboration, and overall project efficiency [1,10]. Studies found that there are numerous opportunities for deploying conversational agents in the AEC industry, including in meetings, briefing, hazard identification, training, collaboration, well-being, facility management, and customer service [1]. The chatbot designed by Tsai et al. [4] provides users with access to a water-related disaster database, an intuitive mobile-device-based user interface, and a user intent mechanism to retrieve the information they need for decision-making. While the chatbot helps users access data easily, it lacks a Natural Language Processing (NLP) algorithm, leading to poor performance with long or natural language inputs. The research [5] that aims to improve the efficiency and user experience of facility management also developed a chatbot that claimed to integrate NLP, Building Information Modeling (BIM), and ontological techniques to increase the efficiency of information retrieval. However, the natural language processing in this study is based on keyword matching that lacks machine learning algorithms, which limits its ability to generate new responses, and there was also a high demand for manual input to maintain the corpus. Lin et al. [11] present an approach for improving data retrieval and representation in cloud-based BIM systems using NLP techniques that can extract relevant data from textual descriptions of building elements in the format of Industry Foundation Classes (IFCs). With the limited capabilities of self-implemented NLP, however, this study only supports simple sentences that contain no verbs or complex calculations, which limits the applicability of the proposed method. Cho et al. [6] proposed a chatbot-assisted construction daily report data management system, which collects conversations among subcontractors on instant messaging applications and then automatically generates daily reports for general contractors. This study adopted the Natural Language Understanding (NLU) capability provided by IBM’s Watson Assistant to establish an NLU model that can understand the intents behind users’ messages for subsequent automatic extraction of the necessary information. This study took advantage of NLU to relieve the burden of system implementation that was encountered by previous studies while maintaining the usability of software applications by taking users’ natural language as inputs. However, the developed chatbot only processed numbers and texts without involving in BIM data retrieval, which has been playing a more and more important role in assisting construction site workers in the last decades.

Those aforementioned studies [4,5,6] have leveraged instant messaging platforms that have been popular in public to enhance the ease of use and usability of information systems; however, most chatbots developed by those studies still rely on a form-based user interface that was offered in conventional information systems to interact with users [4,5]. Some studies adopted self-implemented NLP techniques [5,6] to extract information from textual or spoken inputs, but their lack of natural language understanding models limits their systems’ ability and applicability. While a few chatbots have been developed that leverage NLU models [6], those that also retrieve geometric data on buildings that are essential for construction site workers are still rare.

The chatbot developed in this study used NLU technology to identify the intent behind the utterances commonly used by construction site workers as well as the context information associated with these utterances. Fewer finger motions such as key hits and clicks will be required. Conversational AI-based chatbots are expected to discard the form-based interface that requires the user’s finger motions to obtain their input, thus increasing the ease of use and usability of the information system. Moreover, integrated with BIM technologies to provide geometric data on project buildings, the developed chatbot could help construction workers more effectively acquire the information they need for construction management decision-making. This would contribute to maintaining or even improving construction performance under labor shortages.

### 2.2. Natural Language Understanding (NLU)

**Figure 1:**
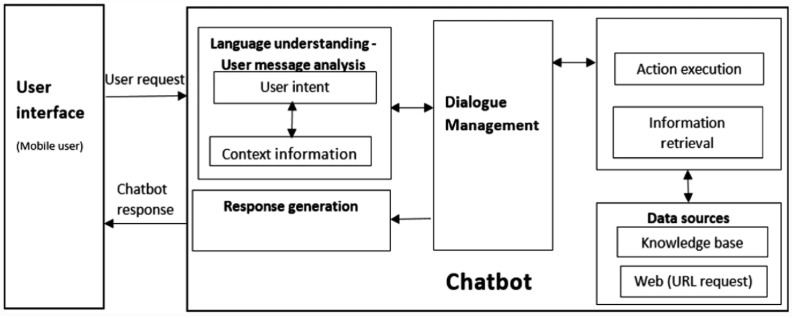
A general chatbot architecture [12].

Figure 1 illustrates the entire architecture of a chatbot deployed on an instant messaging platform [12]. The instant messaging platform provides the user interface between the chatbot and the human user. The mechanism of the chatbot comprises four parts: dialogue management, Natural Language Understanding (NLU), response generation, and data sources, which are described as follows:

1. The dialogue management module: This module is responsible for controlling operational processes among different parts. The main workflow starts with the user message received through the instant messaging platform; then the message is sent to the NLU module for semantic analysis to identify the user’s intent and associated entities. Based on this information, the response system determines a response and replies to the user using the instant messaging platform.
2. The NLU module: This module is responsible for analyzing the user’s messages, where the user’s intent and entities are identified and stored to be processed further. The NLU module comprises an intent classifier and entity extractor. The former analyzes the objective or intent of the user’s message, such as a query, a command, or simply a greeting. The latter extracts the context information in the message, such as the parties in the question, the action in the command, or the emotion conveyed by the message.
3. The response generation module: This module is responsible for determining the responsive action based on the intent and context information identified with the NLU module. Example responses are similar to sending out an order form or writing a record into a database. The response module also determines the response message, which is sent back through the instant messaging platform, thereby completing a cycle of message receiving and replying.
4. The data sources: This part stores sources that can provide answers or responses to the user’s question. It can be a knowledge base, database, documents, or simply web pages.

The essential parts of a chatbot are the NLU module and its dialogue system, which comprises three units: dialogue state tracking (DST), dialogue policy (DP), and natural-language generation (NLG) [10]. In every conversation, the user first provides an utterance, such as “Book an HSR ticket from Taipei to Taichung.” The NLU module analyzes this utterance and identifies the user’s intent—in this instance, booking an HSR ticket—and the context information, or entities, mentioned in the utterance, which are “from Taipei” and “to Taichung”. “Intent” here refers to the user’s purpose, and the “entities” are the objects or parties mentioned in the utterance which constitute the parameters for fulfilling the intent. Next, the DST module determines the current state of the conversation based on the given utterances and the results of prior interactions. This dialogue state is the current entity values based on the dialogue history; for example, the current intent is “booking an HSR ticket”, and the current entities are Taipei as the departure station, Taichung as the arrival station, and a blank field for the departure time and date. The dialogue state is then sent to the DP module, which decides the next action, such as querying the departure time and date. Last, the NLG module generates an appropriate response, such as “When is your travel date?”

Supervised approaches for intent recognition and entity identification require training with large amounts of utterances with identified corresponding intents and entities. For example,

Utterance: Order an HSR ticket from Taipei to Taichung.

Intent: Order an HSR ticket.

Entity (from): Taipei.

Entity (to): Taichung.

**Figure 2:**
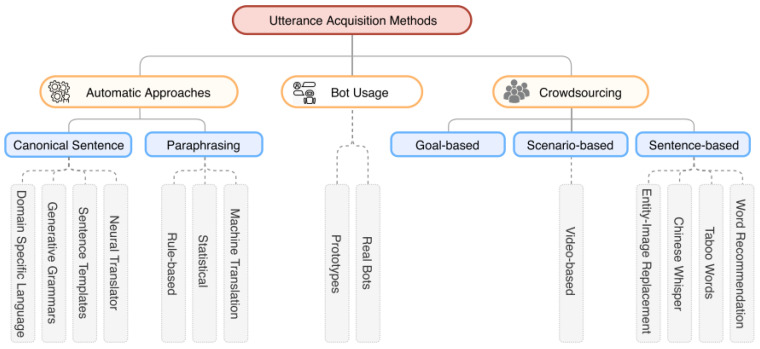
Approaches for acquiring intent recognition model training datasets [14].

The training dataset comprises a set of user utterances with corresponding intents. Given the richness of human natural languages, the same intent can be expressed in various ways. Consequently, numerous utterances with labeled intents and entities are essential for effectively training NLU models. However, building this training dataset is expensive; each intent requires entering numerous utterances in various ways, and the intent and entities in each utterance must be manually labeled [10,13]. According to the literature, three approaches are commonly used to effectively build training datasets for NLU models, as shown in Figure 2:

1. Bot usage: A NLU model is initially trained with few utterances; a chatbot prototype is then given to users who can directly help label the utterances. This is the most direct and convenient approach, and the recognition accuracy will grow over time. However, the labeled utterances must be validated and checked for grammar and spelling errors.
2. Automatic approaches: Automatic approaches comprise two major steps: (1) canonical utterance generation and (2) automatic paraphrasing. Canonical utterances are imperative sentence templates that must contain “placeholders”. An example of a canonical utterance is “Please book an HSR ticket to [destination],” where [destination] is a placeholder. At this stage, a set of canonical utterances needs to be generated to provide several allowable values for placeholders enabling the automatic process to automatically generate the remaining allowable values. Next, automatic paraphrasing relies on the machine’s translation technology, such as rule-based machine translation, statistical machine translation, or neural machine translation, to rephrase the utterance.
3. Crowdsourcing: Labeling and validation are both performed by volunteers on the Internet.

This study aims at demonstrating the concept of conversational AI-based chatbots introduced for construction site managers. Therefore, we only built a comparatively small training dataset; thus, the first approach was used to establish our NLU model.

The rise of chatbots has led to both industry and academia rushing to create NLU platforms that enable anyone, even those without expertise in natural language, to rapidly develop conversational AI-based applications including chatbots. Some popular commercial NLU platforms include Dialogflow by Google, LUIS by Microsoft, Watson Assistant by IBM, and Rasa, which are briefly introduced below:

1. Rasa: Rasa is an open-source machine-learning framework often used for chatbot development. The two main Rasa modules are Rasa NLU and Rasa Core; the former is used to understand the intents and entities in the users’ utterances, and the latter is used in dialogue management.
2. Watson: Watson is a conversational AI platform that can provide developers with accurate answers across applications and devices. It can learn from conversations with users to increase its immediate problem-solving abilities, eliminating the frustrations of long waits and tedious searches.
3. Dialogflow: A SaaS-based conversational AI software from an eponymous company that offers freeware and online support. It also offers developers features such as multiple languages, all channels, on-screen chats, and voice recognition.
4. LUIS: LUIS, which stands for Language Understanding Intelligent Service, is a cloud service based on machine learning that can incorporate natural language into mobile applications, chatbots, and IoT devices. LUIS is designed to identify valuable information in conversations to determine user intent and entities to develop high-quality, detailed language models by offering powerful development tools, customizable prebuilt applications, and an entity dictionary that enables solutions to be built and deployed rapidly. The entity dictionary is mined from web pages and several billion entries that can help users accurately identify valuable information from a conversation are available.

**Figure 3:**
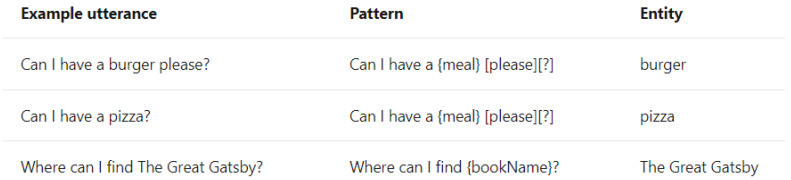
Examples of Pattern.Any Entities [15].

In some domains, one simple intent may involve multiple entities. For example, the utterance “Check balance in my savings account ending in 4406” contains two entities, accountType and accountNumber. In contrast, buying tickets may even require at least four entities: ticketsCount, departureDate, Departure, and Destination. In response to these complexities, Microsoft LUIS offers various entity types [15]:

1. Simple Entity: The individual entities that were previously mentioned are simple entities.
2. Hierarchical Entity: For example, in other scenarios, Location may be a simple entity, but in a ticket booking scenario, it will lead to two Child Entities, namely Departure and Destination. Location is therefore a hierarchical entity, and the algorithm will divide the entity into Location::Departure and Location::Destination for clarity during model training.
3. Prebuilt Entity: LUIS has many common built-in entities such as date, time, numbers, age, currency, and dimensions. Dimension entities associated with construction include length, area, and volume. These entities are prebuilt in the LUIS system and do not require additional manual labeling, thereby greatly reducing the burden of model training.
4. Composite Entity: During the model training process, each labeled entity is stored individually in the training dataset. However, if an utterance is long or contains many associated entities, the entities are labeled as composite entities, and LUIS will gather these entities together to accelerate the training speed and increase recognition accuracy. This mechanism is useful when dealing with a site manager’s utterances, which frequently require the use of composite entities—such as Grid Position, Component’s Family, and Type—to describe a building component.
5. List Entity: Conceptually, a List Entity is a synonym. Some construction terms have various expressions or abbreviations, such as “reinforced concrete structure”, and its abbreviated form, “RC structure” will be in the same entity list.
6. Pattern.Any Entity: A Pattern.Any Entity follows a specific formula and can be applied in sentences with fixed vocabulary and structure. The utterances in Figure 3 all contain examples of Pattern.Any Entities. This feature was helpful when building an NLU model in this study where some complex utterances would appear.

Considering its powerful capabilities and flexibility, Microsoft LUIS was adopted in this study to build the NUL model. The establishment of the model and its implementation will be addressed in Section 3.2.1 and Section 4.1, respectively.

## 3. Approach

**Figure 4:**
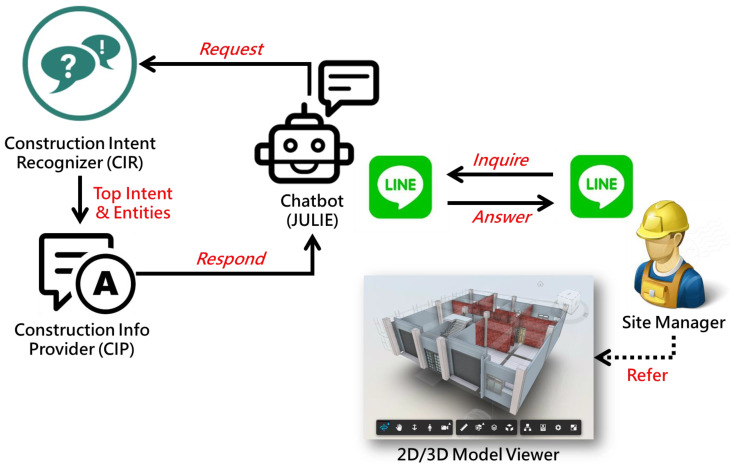
The question–answering process of the chatbot JULIE.

This section introduces two core components inside chatbot JULIE: (1) *Construction Intent Recognizer (CIR)*, the NLU model for understanding the question and (2) *Construction Information Provider (CIP)*, the processor consisting of disparate algorithms for finding the answer. Figure 4 illustrates the relationship and cooperation between the two components. The approach to developing these components is also described.

### 3.1. Overview

The question–answering process of chatbot JULIE, similar to the human brain’s operation, consists of two phases: (1) understanding the question and (2) finding the answer. In order for the Construction Intent Recognizer (CIR) to understand questions about the building component’s dimension, a semantic framework of inquiries, in the form of “utterances” grouped by “intents”, is built in advance. These “utterances” include those common question expressions, both formal and informal, with the same “intent” raised by site managers or field engineers for their daily work. Each utterance could be labeled with one or more “entities”. Thanks to the advances in Natural Language Processing (NLP) in recent years, only a couple of utterances for an intent are needed and used as training and testing cases to create an NLU model that can recognize the intent behind utterance as well as identify entities used with this intent. Once established, the NLU model can be requested by chatbot JULIE to predict both intents behind questions and entities enclosed in that question. The top intent with the highest prediction confidence and entities used in that intent will be passed to the Construction Information Provider (CIP), a processing module embedded with groups of algorithms for finding the answer. The establishment of CIR will be elaborated on in Section 3.2 while CIP will be introduced in Section 3.3.

### 3.2. Construction Intent Recognizer (CIR)

As mentioned earlier, the Construction Intent Recognizer (CIR) is a trained NLU model that can understand questions by predicting the intent and the entities used in that intent. It begins with establishing a semantic framework where the intents of inquiries about the building component’s dimension are first defined, followed by manually specifying the entities that are used in intents. Next, the model undergoes a process of training and testing to become a trained model, which is ready to be deployed for chatbot JULIE to request. The method to define intents and label entities is described in Section 3.2.1, while the learning process of the NLU model is described in Section 3.2.2.

#### 3.2.1. Intent Definition and Entity Labeling

**Figure 5:**
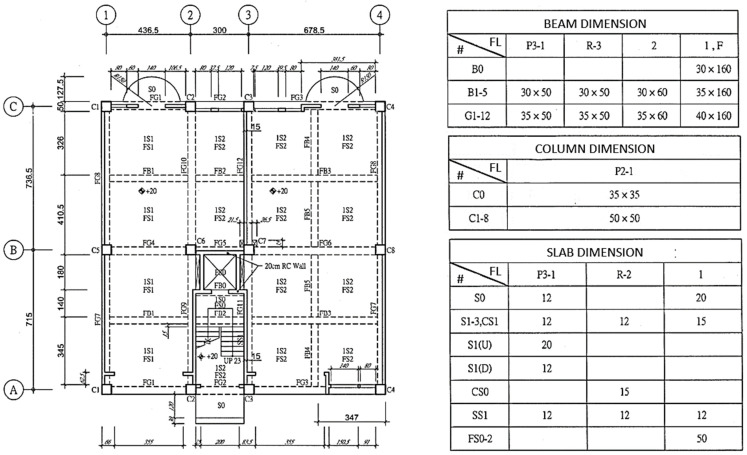
Examples of dimensions for individual components.

**Figure 6:**
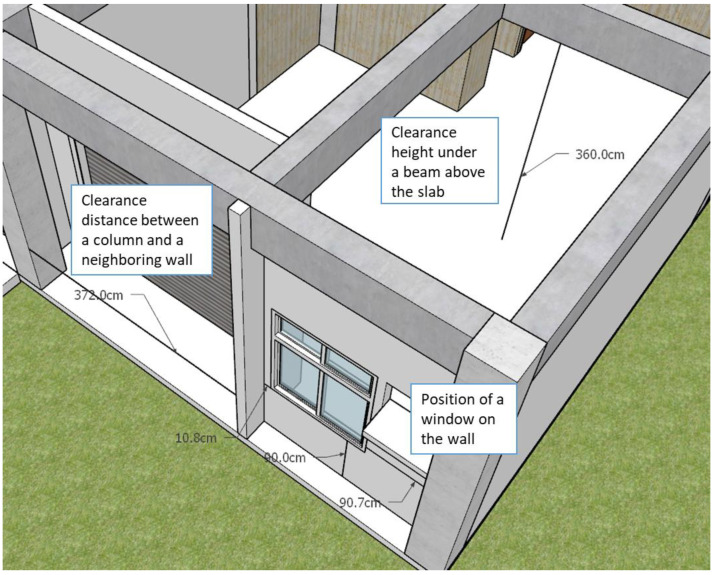
Examples of spatial relationship among two or more components.

Intents behind those inquiries about building component dimensions by site managers for their daily routines in construction sites can be roughly classified as followings:

- Baselines: includes vertical baselines, such as floor lines, and horizontal baselines, such as column center lines, as the circled letters and numbers shown in Figure 5, or wall center lines.
- Dimension for individual components: For example, the width and depth of beam B3 on Level 2, the thickness of slab CS1 on Level 3, or the height of a certain floor, as the dimension tables illustrated in Figure 5.
- Spatial relationships among individual components: For example, the position of a window on the wall, the clearance height under a beam above the slab, the distance between two column centers, or the clearance distance between two neighboring walls, as illustrated in Figure 6.

In order to obtain the accurate and precise dimension that site managers request, utterances for all intents mentioned above should be exactly specified with component reference, i.e., which component is under inquiry. In general, there are three common ways to specify a component of a building: (1) named method; (2) grid method; (3) georeferenced method, and (4) hybrid method. For architectural projects, all structural components and most architectural components would be given with a name or label in drawings, as shown in Figure 5. Thus, for the named method, site managers can easily refer to a specific component using those names or labels when they are aware of the one for the component they inquire. However, this could not be the case when site managers just stand in front of the column or under the beam inside the building. For most cases without being aware of component labels, site managers can refer to a component using XY grids, for example, Column C6 in Figure 5 can be referred to as the column from Grid B to Grid 2; or Beam FG3 can be referred as the beam on Grid A from Grid 3 to Grid 4. Those grid numbers can be identified by counting the columns in the building. In both the named method and grid method, component references for building projects usually include the floor or level number in order to be precise. The georeferenced method is based on the named method or grid method and refers to components by introducing a spatial relationship such as an intersection or boundary touching, within a distance, which is more complex and beyond our study’s scope.

The other category of intents involves two or more components in one single utterance. They often come in a spatial relationship among neighboring components. For example, the clearance floor height under a beam, as shown in Figure 6, is the distance between the beam’s bottom and the top of the slab under the beam. A complex intent is the position of a window on the wall where several distances should be specified.

Table 1 summarizes some of intents and utterances with their entities for dimension of individual components using both named method and grid method, respectively. These intents in Table 1 only include single component, and thus are much easier for CIP to implement the algorithms for finding answer. Therefore, in this study we only implemented intents for the dimension of individual components in order to demonstrate and verify our ideas. Intents with complex relationships will be further studied in the future.

#### 3.2.2. Learning Process and Deployment

**Figure 7:**
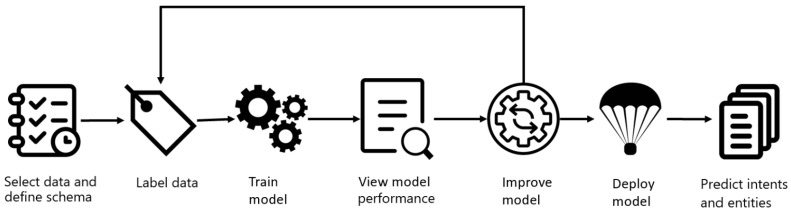
Learning process for the NLU model [15].

Figure 7 describes the learning process of the NLU model. First, utterances of all three pre-defined intents and “None” are split into a training set and testing set in a user-defined proportion. Next, the model enters a cycle of learning and improving, where the prediction performance is evaluated in each cycle to determine if new utterances need to be included or entities need to be modified. Once the model performance is satisfied, it can be used to predict intents and entities in the real world.

During the performance evaluation, the testing set is used to calculate the confusion metrics, which report the counts of the true positive, true negative, false positive, and false negative predictions of the model. Three indicators derived from the confusion matrices are calculated to evaluate the performance, including precision, recall, and f1-score, which are defined according to Formulas (1)–(3), respectively. Precision measures how accurate a model is. It shows how many of the predicted intents and entities are correctly labeled. Recall measures the model’s ability to predict actual positive classes. It is the ratio between the predicted true positives and what was actually labeled. The recall indicates how many of the predicted classes are correct. The f1-score is also a measure of a model’s accuracy on a dataset. It is defined as the harmonic mean of the precision and recall of the model. In this study, precision, recall, and f1 score are calculated for three levels of evaluation: (1) entity-level: metrics are calculated for each entity separately; (2) intent-level: metrics are also calculated for each intent separately; and (3) model-level: metrics are calculated for the model collectively. More details of the performance evaluation will be addressed in Section 5.2.

(1) $$Precision=\frac{\#True\;Positive}{\#True\;Positive+\#False\;Positive}$$

(2) $$Recall=\frac{\#True\;Positive}{\#True\;Positive+\#False\;Negative}$$

(3) $$f1-score=2*\;\frac{Precision*Recall}{Precision+Recall}$$

### 3.3. Construction Information Provider (CIP)

**Figure 8:**
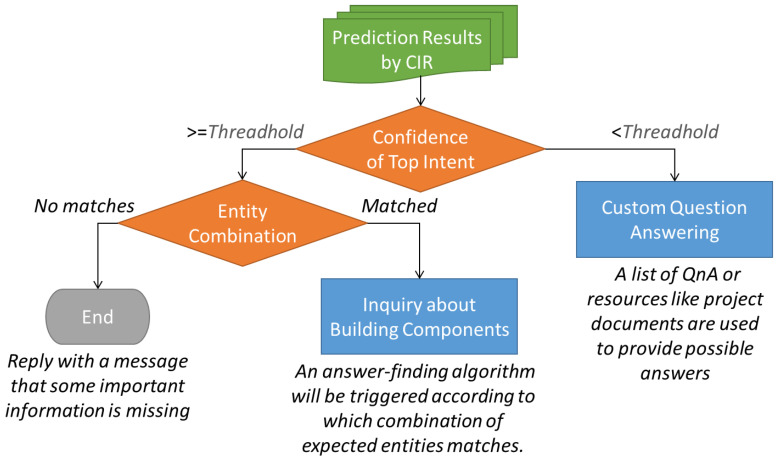
The flowchart of pre-processing with CIP to dispose of the prediction result from CIR.

When requested, *CIR* would give a prediction of each intent defined in the NLU model with confidence. The intent with the highest prediction confidence is specified as the top intent. Those entities used in top intent are also given with confidence if they are identified. These prediction results are then passed to the *Construction Information Provider (CIP)* for finding the answer. However, only the top intent with a confidence higher than a pre-defined threshold, e.g., 80%, will be qualified; then, the *CIP* would initiate a process to determine which answer-finding algorithm will be triggered according to which combination of expected entities matches. This process is introduced in Section 3.3.1. If no combination of expected entities is matched, the *CIP* would directly reply with a finalized message notifying that some important information is missing. For those situations where the prediction confidence of top intent is lower than the threshold, the *CIP* would trigger an alternative process using custom question answering, where a list of questions and answers is extracted from project documents to provide possible answers, which is described in Section 3.3.2. Figure 8 illustrates the flowchart of the aforementioned process using the *CIP* to dispose of the prediction result from the *CIR.*

#### 3.3.1. Response to Inquiry about Building Components

**Figure 9:**
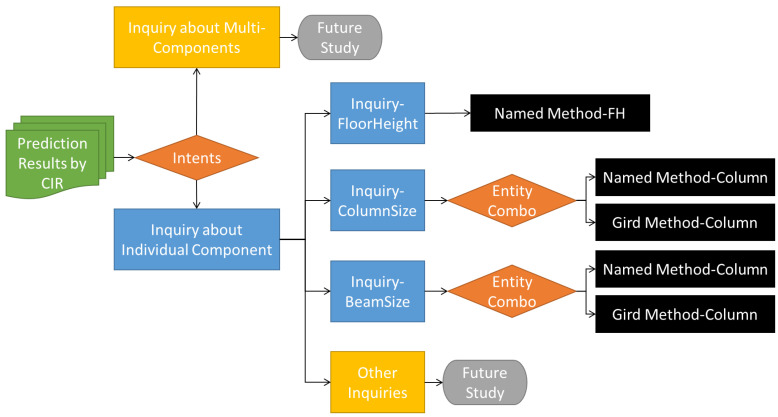
Disposing of inquiries about building components with matched entity combinations using the CIP.

Figure 9 illustrates the disposal of responding to those inquiries about building components with matched entity combinations by CIP. The scope of inquiries should include both individual components and multi-components as mentioned in Section 3.2.1. However, this study aims at demonstrating the idea of a chatbot used on construction sites, and therefore merely considers those individual components. Moreover, three categories of those inquiries most commonly seen in construction sites, including inquiries about floor height, column size, and beam size, were first researched and implemented.

Under each category, there exist one or more sub-categories of the disposing algorithm according to their matched entity combination. For example, an inquiry about column size may contain either the column label (Named Method) or grid labels (Grid Method), where different algorithms would apply accordingly. For all categories of inquiries, the georeferenced method and hybrid method for component reference could also be used; however, they are less seen in reality and much more complicated to analyze and implement, and thus are beyond the scope of our current study.

In the era of traditional 2D drawings, there were few options except reading drawings for site managers to obtain the dimension of building components. With the advance of 3D modeling techniques, or Building Information Modeling (BIM), in the last decade, they can also make use of 3D building models to access component information they need. Likewise, algorithms implemented by the CIP also take advantage of BIM to make it much easier to find the answers to those inquiries about component dimensions.

In general, the typical way for software programmers to automatically inquire about component dimensions from 3D building models is to parse their spatial topology. Spatial topology is the 3D data model that represents the geometric relationships among building components using mathematical notation or digital data structure. Some spatial topologies commonly seen in the A/E/C industry include IFC, STEP, IGES, OBJ, and STL. All 3D modeling software would have its own spatial topology to represent 3D objects as well as provide an interface to export to other data formats for data exchange.

**Figure 10:**
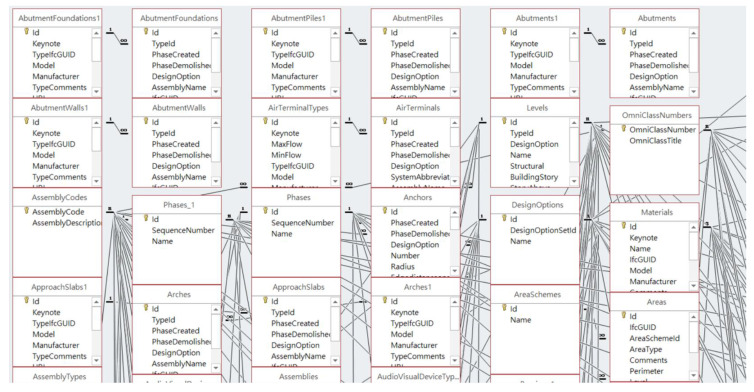
An example of the ODBC database exported from Autodesk Revit.

However, despite the fact that spatial topology can provide all kinds of geometric information for any building model, it usually requires a great amount of energy and effort for software programmers to understand the complex data schema in order to parse and manipulate it. Instead, this study takes advantage of one of BIM’s capabilities that can customize and represents the model’s attribute and geometry in a tabular schema to implement the algorithms of CIP. For example, a commonly used BIM software, Autodesk Revit, provides the capability to export a 3D building model to the ODBC database, which contains more than 300 tables with attributes related to structural and architectural components, as shown in Figure 10. Those tables preserve basic information about components. For example, Table *StructuralColumns* contains attributes such as *FamilyName*, *TypeName*, *SectionDimension*, *StructuralMaterial*, *TopLevel*, *BaseLevel*, Volume, and *ColumnLocationMark*, which provide the sources for responding to inquiries about building components by site managers.

**Figure 11:**
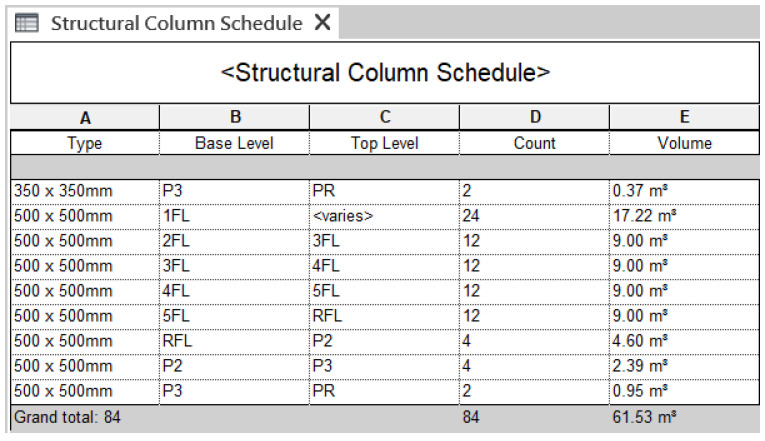
An example of a customized schedule provided by Autodesk Revit.

BIM software also produces another tabular called schedule, as shown in Figure 11, which is extracted from the properties of building components. Site managers can customize schedules with more useful and aggregative information, and export them to another software program, such as a spreadsheet, which provides an easier way to retrieve information. This study also adopts customized schedules as a source for CIP to collect answers to a site manager’s inquiries.

#### 3.3.2. Custom Question Answering

In addition to the component dimensions, site managers also require other project information for their management tasks such as contract, bill of quantities, schedule, cost estimate, and progress reports. These types of information are distributed among all kinds of project documents. Although mainly focusing on responding to inquiries about component dimensions whose data source is drawings or building models, this study still preserves the capability of chatbot JULIE to respond to inquiries other than component dimensions, which would be implemented in the future.

As mentioned earlier, for those Top Intent with a prediction confidence lower than the thread hold, CIP would trigger custom question answering to provide alterative responses other than dimensions. Custom question answering is the previous generation of natural language understanding used to find the most appropriate response for utterances from the custom knowledge base containing question and answer pairs. The custom knowledge base can come in structured sources such as spreadsheets, documents, and Portable Document Format (PDF) as well as unstructured sources such as web pages.

In order to demonstrate the ability of custom question answering, we prepare several project documents as the knowledge base, which come in formats including MS Word (*.doc) and Portable Document Format (*.PDF). More details will be introduced in Section 4.2.

## 4. Chatbot Prototyping

**Figure 12:**
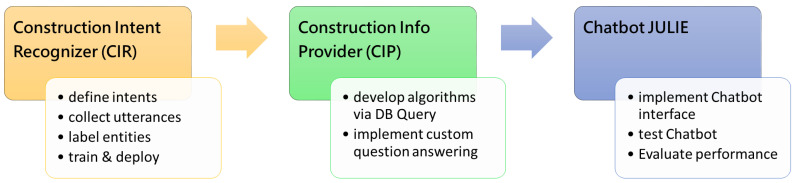
Steps of implementing CIR, CIP, and chatbot JULIE.

Because of an extraordinarily high market share of 95% in Taiwan, LINE, the most popular mobile instant messaging platform was chosen for this study to implement and deploy the chatbot JULIE using LINE Messaging APIs. Figure 12 illustrates the sequence of implementing the entire system of chatbot JULIE, including three major modules: *Construction Intent Recognition (CIR)*, *Construction Information Provider (CIP)*, and the main body of the chatbot. The details for the implementation of each module are described in the following sections.

### 4.1. Construction Intent Recognition

Ideally, the application scope of a chatbot for site managers would cover intents as broad and complex as possible. This study, however, aims to demonstrate how the idea of introducing a chatbot can benefit site a manager’s daily work; therefore, we only collect some common intents and utterances with a high frequency of use by site managers.

This study used Microsoft LUIS to build the NUL model in the conceptual design phase, and during the phase of prototyping, we migrated to the next generation of LUIS, Conversational Language Understanding (CLU), which is currently a feature of Azure Cognitive Service for Language. The core mechanism of the two generations and ways to establish the NLU model are mostly identical.

Table 2 illustrates the intents and utterances along with their labeled entities collected in this study for demonstration using Microsoft LUIS, which provides an easy-to-use interface to manage entity labeling. Those underlined terms in Table 2 are labeled entities with their entity names specified below. The meanings of those utterances can be found in Table 1 and Figure 5 and Figure 6.

**Figure 13:**
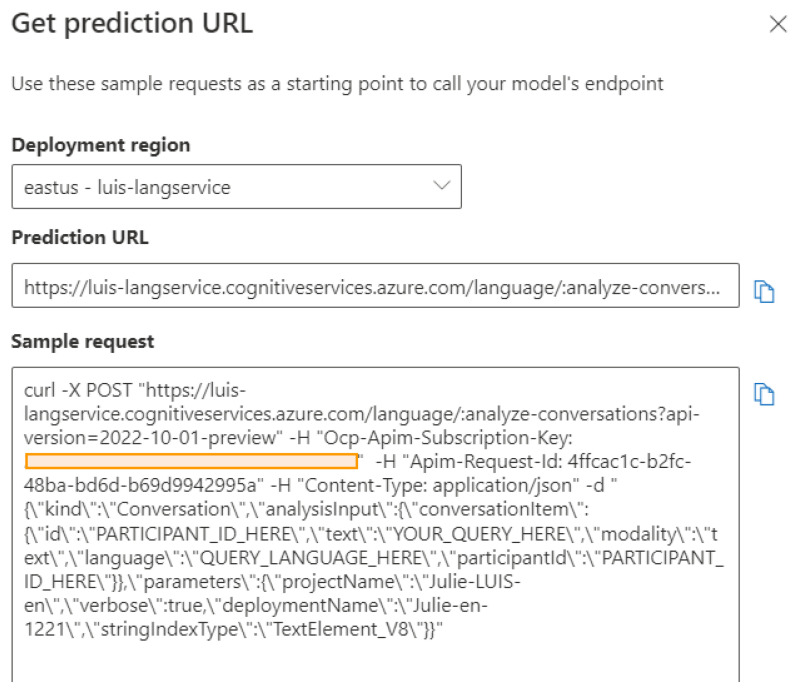
The production URL generated with Microsoft LUIS for end users to call.

During the model training, all utterances with pre-defined intents and labeled entities are split into a training set and testing set in a 64:36 proportion. In each training cycle described in Section 3.3.2, the prediction performance is evaluated to determine if new utterances need to be included or if entities need to be modified. A complete training process for a successful NLU model requires several training cycles by adding or removing training cases according to the testing results. Once the prediction performance is satisfactory, the model can be deployed and a production URL will be generated for end users to call, as shown in Figure 13. Section 5 will exhibit a training cycle to demonstrate the effect of progressive improvement during the model training.

Despite the fact that our demonstrative NLU model contains merely some intents and utterances, the research team still drew a progressive action plan as follows for future implementation because the method to collect and label utterances would differ with the number of utterances and complexity of the entity.

1. Collection and extraction of massive utterances: In order to collect massive training cases, site managers can wear a voice recording device during their regular coordination meetings. Next, voice-to-text software can be used to convert the sound files into text, followed by a procedure to automatically extract utterances.
2. Semi-automatic entity labeling: The most time- and energy-consuming entity annotation can be alleviated by applying both automated procedure and labor work to label entities in order to minimize manual efforts. Next, those labeled entities can be verified using crowdsourcing. The error cases are fed back to the model training process, thus improving the prediction accuracy.

### 4.2. Construction Information Provider

**Figure 14:**
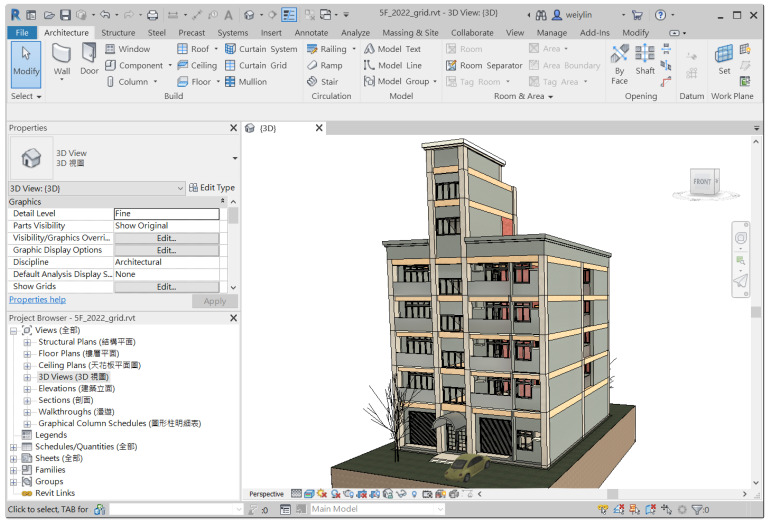
A residential building modeled using Autodesk Revit is used to implement the *CIP* algorithms.

As mentioned in Section 3.3.1, this study only considered three commonly seen intents of inquiring about individual components, and thus only corresponding algorithms dealing with those intents and their matched entity combination were implemented. As shown in Figure 14, a residential building of five floors modeled with Autodesk Revit was used to implement the disposing algorithms of the CIP. Instead of directly parsing the spatial data model of the Revit model, this study takes advantage of BIM’s capability to customize and represent a model’s attributes and geometry in a tabular schema, and, therefore, algorithms were implemented based on a relational database exported from Revit. Section 4.2.1 and Section 4.2.2 describe the algorithms dealing with inquiries about the floor height and the dimension of structural columns, respectively.

#### 4.2.1. Algorithm for Inquiring about the Floor Height

**Figure 15:**
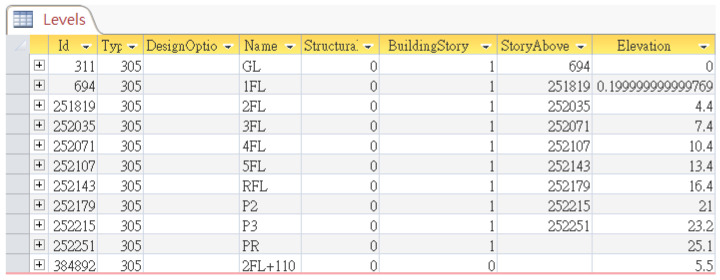
Table “*Levels*” and its fields in the model database exported from Autodesk Revit.

**Figure 16:**
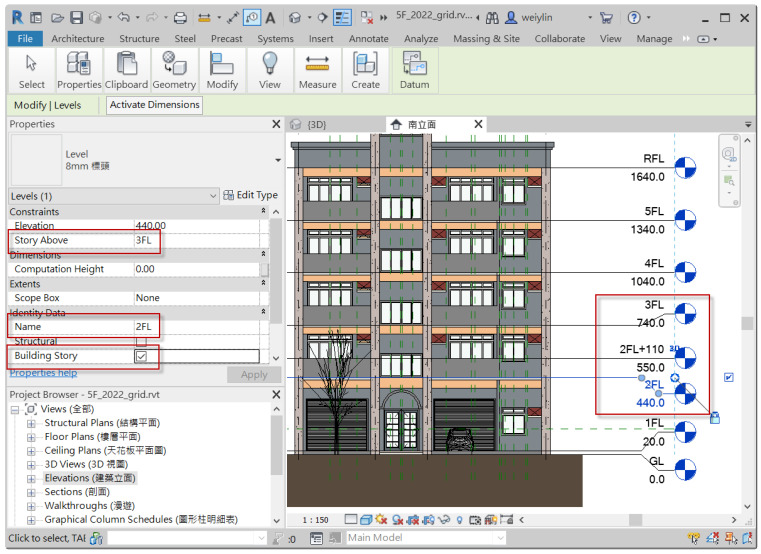
Illustration of Field “*StoryAbove*” of Table “*Levels*” exported from Autodesk Revit.

The relational database exported by Autodesk Revit contains a table “*Levels*” which represent finite horizontal planes that act as a reference for level-hosted elements, such as roofs, floors, and ceilings. This table provides basic information of all levels specified in the Revit model, whose table fields include *Name*, *BuildingStory*, *StoryAbove*, *Elevation*, and so on, as shown in Figure 15 [16]. *BuildingStory* indicates whether or not a level corresponds to a functional story or floor in the model, while “*StoryAbove*” indicates the next building story for the level [16]. By default, the value of *StoryAbove* is the next highest level for which Building Story is enabled, but this is not always the case. Referring to Figure 16, the “*StoryAbove*” the building story “2FL” (elevation = 4.4 m) should be “3FL” (elevation = 7.4 m), instead of its next highest level “2FL+110” (elevation = 5.5 m).

**Figure 17:**
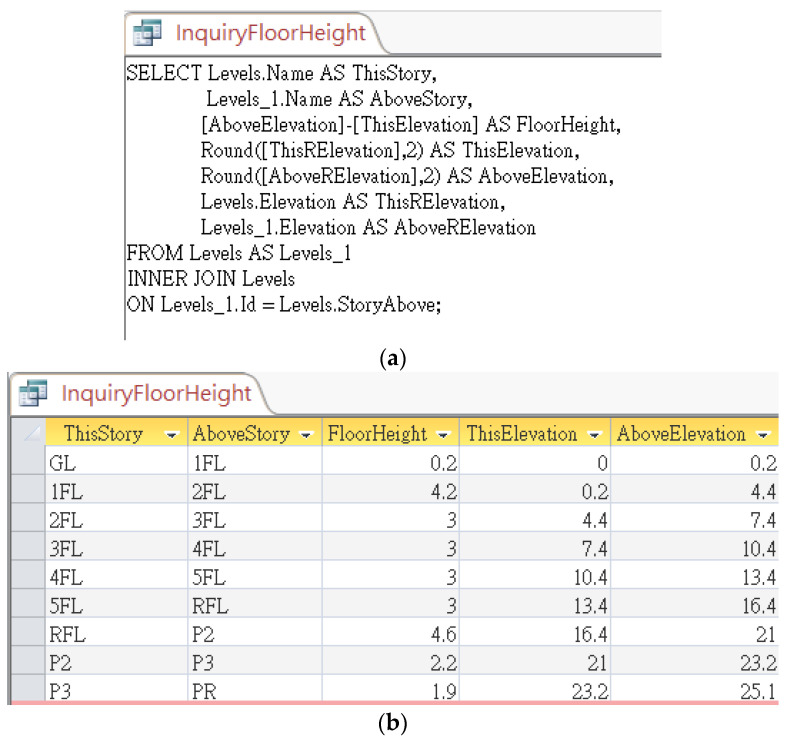
Pre-defined query for responding to an inquiry about the floor height: (**a**) SQL syntax, and (**b**) query results.

Normally, the modelers do not need to specify *StoryAbove* for each level when modeling. However, in order to make good use of this database for algorithm implementation, the research team manually specified these values in Revit, exported them to a database, and created a pre-defined query, as shown in Figure 17a, to calculate the floor height for each story. The query results are shown in Figure 17b. This query can then be requested using the CIP algorithm by applying the following SQL statement feeding with a parameter of *FloorName* predicted with the CIR previously:

**SELECT** *FloorHeight*.

**FROM** *InquiryFloorHeight*.

**WHERE** *ThisStory = [FloorName]*.

#### 4.2.2. Algorithm for Inquiring about the Dimension of Structural Columns

**Figure 18:**
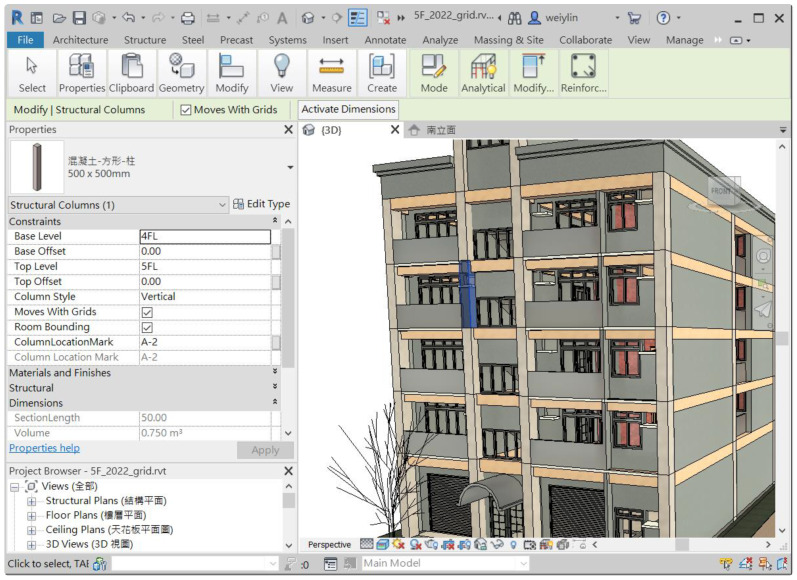
Illustrations for attributes of the database table “*StructuralColumns*” exported from Autodesk Revit.

**Figure 19:**
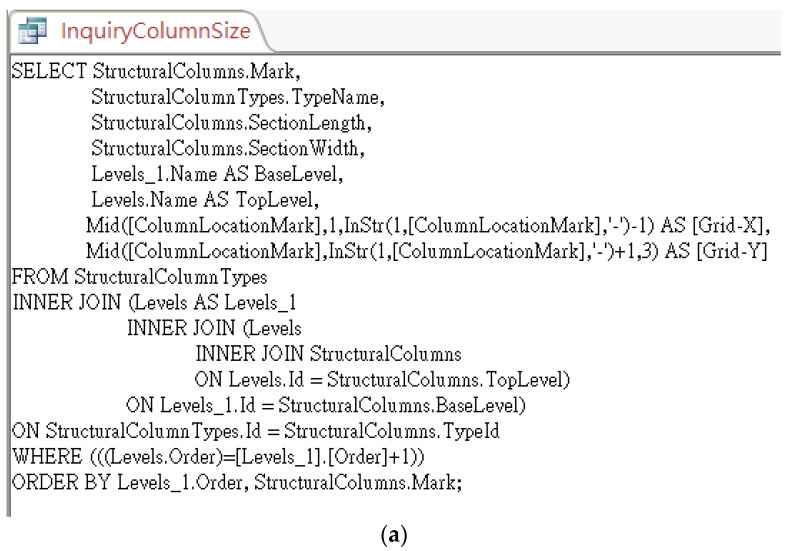

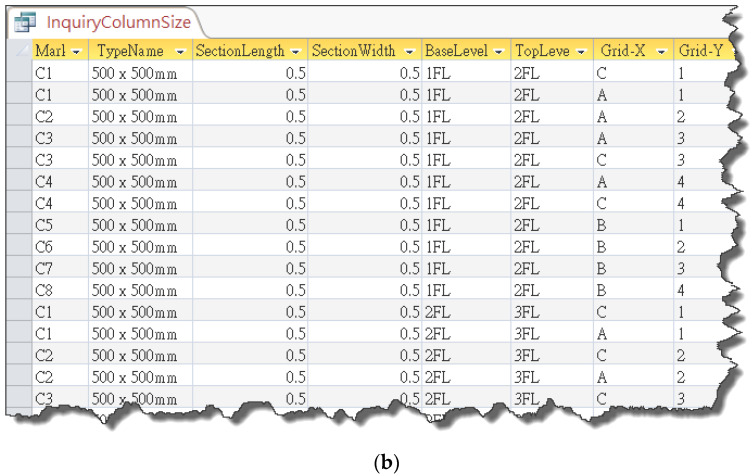
Pre-defined query and results for responding to an inquiry about the size of structural columns: (**a**) SQL syntax and (**b**) query results.

Similarly, we also make use of the table exported from Revit to implement the algorithm for inquiring about the dimension of structural columns. Referring to Figure 18, the table *StructuralColumns* contains fields such as *FamilyName*, *TypeName*, *BaseLevel*, *TopLevel*, and *ColumnLocationMark*, where *BaseLevel* and *TopLevel* indicate level constraint for a column, while *ColumnLocationMark* specifies the coordinate location of a column on project grids [17]. This column information is useful to create and pre-defined query and thus ease the implementation of the algorithm for dealing with inquiries about column size either using a column’s label (Named Method) or using grid labels (Grid Method). Figure 19a is the pre-defined query for responding to inquiries about the size of structural columns, while Figure 19b shows the query results.

With the pre-defined query, as shown in Figure 19, the algorithm can deal with those inquiries using the named method and may apply the following SQL statement feeding with a parameter *FloorName* and *ColumnLabel* predicted with CIR previously:

**SELECT** *SectionLength, SectionWidth*

**FROM** *InquiryColumnSize*

**WHERE** *BaseLevel* = *‘*[FloorName*]’*
**AND**

*Mark* = *‘*[ColumnLable*]’*

Likewise, the SQL statement for those inquiries using the grid method is shown below:

**SELECT** *SectionLength, SectionWidth*

**FROM** *InquiryColumnSize*

**WHERE** *BaseLevel* = *‘*[FloorName*]’*
**AND**

*((Grid-X* = *‘*[Grid-X]*’*
**AND**
*Grid-Y* = *‘*[Grid-Y]*’)*
**OR**

*(Grid-X* = *‘*[Grid-Y]*’*
**AND**
*Grid-Y* = *‘*[Grid-X]*’))*

## 5. Evaluation and Discussion

### 5.1. Evaluation

This section presents two training cycles with different training datasets to illustrate how to evaluate the prediction performance of an NLU model and demonstrate the effect of progressive improvement during the model training.

**Figure 20:**
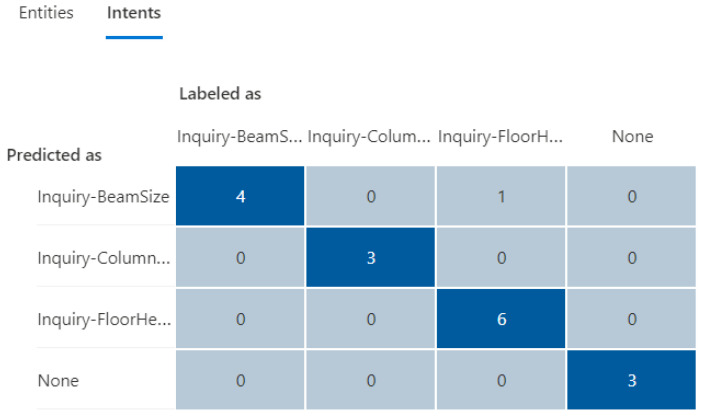
The confusion matrix for intent prediction using the training dataset-215.

**Figure 21:**
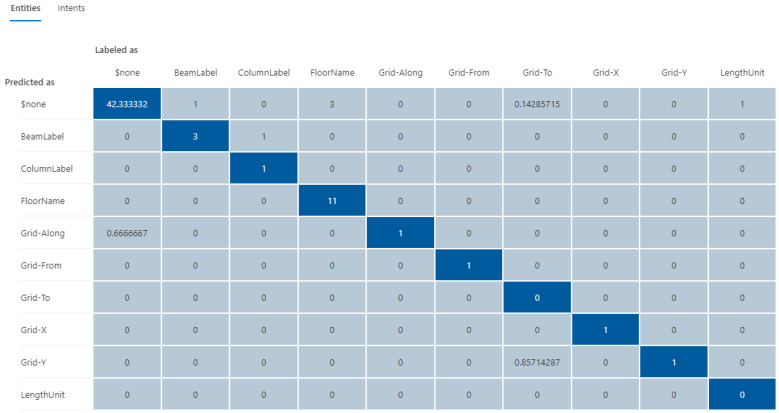
The confusion matrix for entity prediction using the training dataset-215.

The initial training dataset (referred to as “training dataset-215”) contains a total of 21 utterances with 38 entities (as shown in Table 2), including a trivial intent with 5 utterances. The second column in Table 3 and Table 4 presents the distributions in utterances per intent and total entities in the training dataset-215, respectively. Next, a fixed testing dataset with 17 utterances and 26 entities was used to test the prediction accuracy of the NLU model. Figure 20 and Figure 21 display the testing results in confusion matrices for both intent prediction and entity prediction using the training dataset-215, respectively. The precision, recall, and f1-score for intent prediction are 94%, while the figures for entity prediction are 85.7%, 69.2, and 76.6%, respectively. Taking a close look at the confusion matrices and the testing cases, major incorrect predictions occur in the utterances containing “*level*”, which are not available in the training dataset-215. To fix this issue, nine new utterances containing “level” and annotated as “floor name” (as shown in Table 5) were added to the training dataset (referred to as “training dataset-216”). The third column in Table 3 and Table 4 presents the distributions in utterances per intent and total entities in the training dataset-216, respectively.

**Figure 22:**
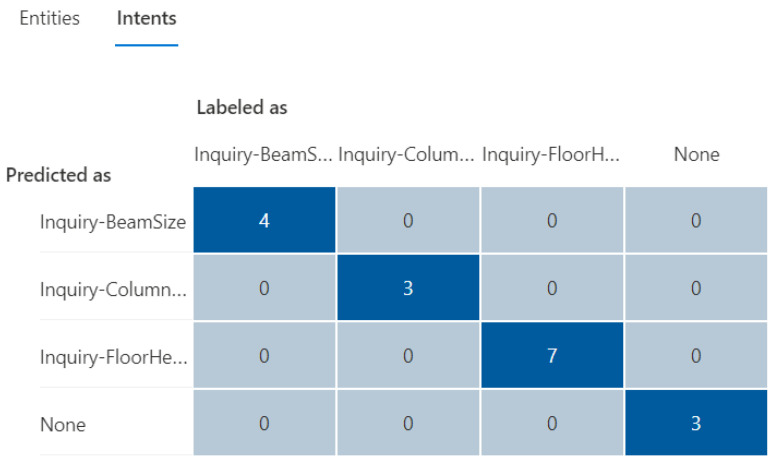
The confusion matrix for intent prediction using the training dataset-216.

**Figure 23:**
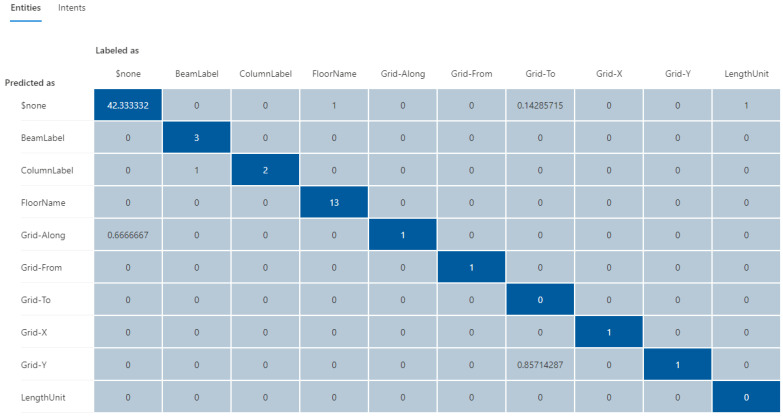
The confusion matrix for entity prediction using the training dataset-216.

The updated testing results using the same testing cases on the training dataset-216 are shown in Figure 22 and Figure 23, demonstrating a significant improvement in both intent and entity prediction with f1-scores of 100% and 84%, respectively. This iterative process can be continued with additional testing cases to further to fine-tune the NLU model until a satisfactory level of prediction accuracy is achieved.

### 5.2. Discussion

As comparable task-oriented chatbots to chatbot JULIE may not be readily available, this section presents a comparison between chatbot JULIE and one of the latest Natural Language Understanding (NLU) techniques, ChatGPT, a non-task-oriented chatbot developed by OpenAI [7]. This is followed by a brief discussion of the research contributions and limitations.

ChatGPT was selected for comparison in this study because both models are designed to process and understand human language input, with the aim of extracting meaning and making predictions based on that meaning. However, there are notable differences between the models. For instance, ChatGPT’s NLU model uses the Transformer architecture, a deep learning model that has achieved state-of-the-art performance in many Natural Language Processing (NLP) tasks. In contrast, LUIS utilizes a combination of machine learning algorithms and rule-based methods to comprehend language. Moreover, ChatGPT’s NLU model is pre-trained on a large corpus of text data, whereas LUIS enables developers to fine-tune the model on their own data.

Nevertheless, as a non-task-oriented chatbot, ChatGPT can only provide general answers based on its vast dataset sourced from the internet, which includes a diverse range of text. It is not capable of interacting with websites or input conditions to inquire about specific information. Consequently, ChatGPT is unable to provide specific answers, such as the floor heights of the building shown in Figure 14, as chatbot JULIE does. As such, the comparison only covers the phase of understanding the question in the chatbot’s question–answering process.

Table 6 presents a comparison of the prediction performance between chatbot JULIE (based on a training dataset-216) and ChatGPT using nine testing cases. As shown in Table 6, chatbot JULIE was able to correctly predict the top intents and their associated entities for eight out of nine testing cases, except for testing cases one–three where the floor name could not be identified. On the other hand, ChatGPT performed very well in all nine testing cases, achieving 100% accuracy in terms of entity prediction. In all nine testing cases, the intent predicted with ChatGPT was “Inquiry”. Given that ChatGPT is constructed using a vast dataset containing billions of training instances, chatbot JULIE can achieve comparable performance with a significantly smaller dataset. This suggests that establishing a self-owned NLU model for a specific application remains a cost-effective approach.

**Figure 24:**
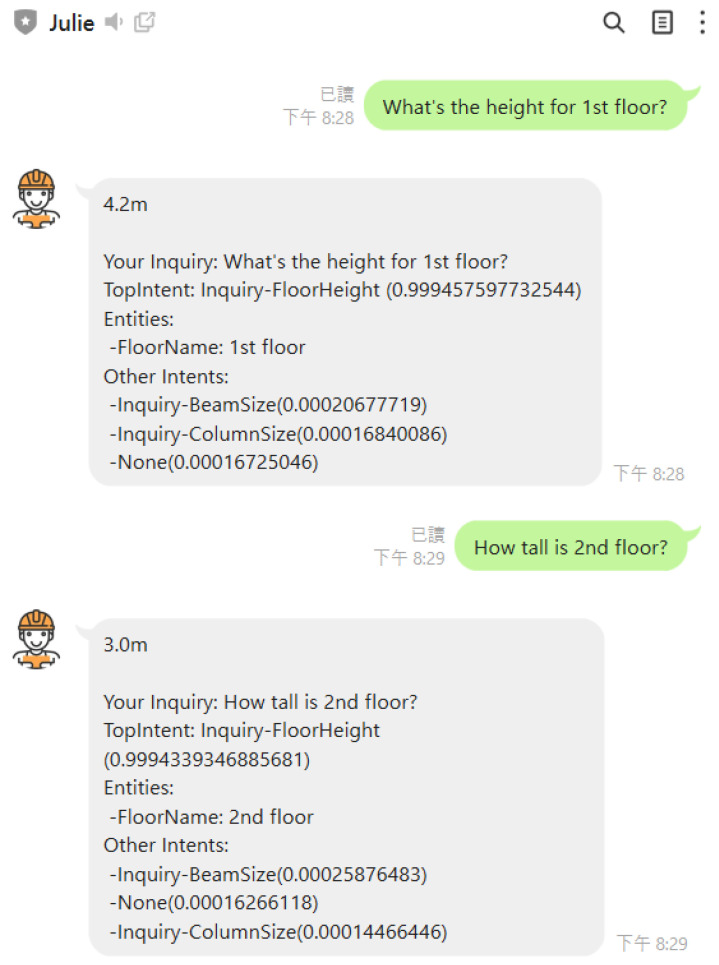
Response examples from chatbot JULIE to inquiries about floor heights.

**Figure 25:**
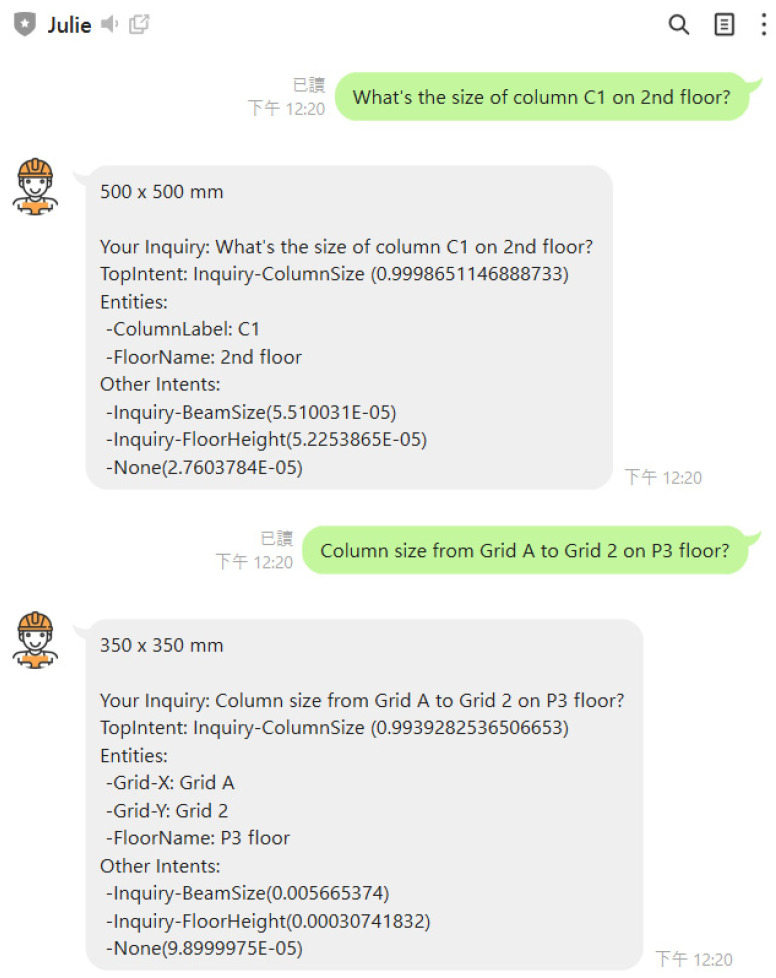
Response examples from chatbot JULIE to inquiries about structural column sizes.

Of particular note, ChatGPT, being a non-task-oriented chatbot, is unable to provide specific answers in the manner of chatbot JULIE. Specifically, in our case, ChatGPT is incapable of retrieving the dimensions of building models, despite its ability to flawlessly predict the intent and entities behind such queries. As illustrated in Figure 24 and Figure 25, chatbot JULIE provides examples of retrieving floor heights and structural column sizes for a particular floor or column. In addition to introducing natural language understanding techniques for chatbot implementation, this study’s principal innovation is the proposition of an alternative method for parsing the spatial data model of buildings. This is achieved by utilizing one of the capabilities of Building Information Modeling (BIM), which involves customizing and representing a model’s attributes and geometry in a tabular schema to implement algorithms for finding answers. This method relieves the burden of chatbot implementation encountered in previous studies.

## 6. Conclusions

The scale and complexity of construction projects are continuously increasing, leading to a growing demand for construction site workers and managers to access information more accurately and efficiently, in order to make informed decisions. Given its high portability, the smartphone has become the most practical and popular device for these users to access work-related data. However, due to its limited screen size compared to PCs or laptops, the usability of software applications on smartphones is of paramount importance. Conversational AI, also known as a chatbot, offers an opportunity to enhance the software usability by allowing for open-ended questions.

This study proposed a two-phased framework for developing the aforementioned chatbots, which includes (1) understanding the question and (2) finding the answer. In phase #1, three categories of intents behind the inquiries made by construction site managers was identified including (1) baselines, (2) dimensions for individual components, and (3) spatial relationships among individual components. A demonstrative Natural Language Understanding (NLU) model was built using Microsoft Azure Cognitive Service for Language to understand the question. To facilitate future implementation in the field, a progressive action plan was suggested to collect and extract massive utterances, as well as to adopt semi-automatic entity labeling to increase prediction accuracy. In phase #2, an alternative method was proposed to parse the spatial data model of buildings using one of BIM’s capabilities that can customize and represents the model’s attributes and geometry in a tabular schema to implement the algorithms for finding the answer.

A prototype of a chatbot named JULIE, based on the aforementioned approach for site managers to inquire about building component dimensions, was developed and deployed using LINE Messaging APIs. The preliminary testing results showed that the chatbot JULIE can successfully predict the intents and entities embedded in the inquiries raised by site managers with satisfactory accuracy, both for intent prediction and for answering the original question. This indicates that our concept can work well and fulfill the research objectives. Additionally, this study presented a novel method to ease the burden of implementing algorithms for chatbots to obtain building component dimensions using BIM techniques, instead of directly parsing 2D drawings or 3D spatial data models.

The proposed approach can be applied to many other tasks for workers whose daily routines heavily rely on mobile phones to retrieve information, instead of devices with large screens, such as personal computers and laptops. Examples in the construction industry include project managers, on-site engineers and architects, inspectors, safety managers, and equipment operators. For other industries, there will be even more examples such as delivery couriers, sales representatives, journalists and reporters, healthcare professionals, retail workers, transportation workers, emergency responders, and so on.

However, the proposed NLU model has a weakness in that the classification of possible intents behind inquiries about building component dimensions is based on personal knowledge and experience, which may not be sufficient and complete. There may be other perspectives to classify possible intents, resulting in different models. Additionally, the classification may be subject to the management styles of site managers from one construction firm to another. Therefore, a generalized hierarchy or framework of intent classification is needed for future research. While additional testing and research efforts are required in the future, it can be concluded that utilizing conversational AI and BIM has the potential to alleviate the shortcomings of traditional database applications, providing site workers and managers with a more effective means for retrieving the information they require.

## Figures and Tables

**Table 1:** Intents and utterances with their entities for the dimension of individual components.

Intents	Labeled Utterances		Entities Used with This Intent
	Named Method	Grid Method	
Inquiry: FloorHeight	How tall is the 2nd floor? What’s the height of level 3? What’s the floor height for B1?	N/A	FloorName, LengthUnit
Inquiry: ColumnSize	What’s size of Column C1 on 1st floor? What’s the dimension for Column C3 on level 2?	What’s the size for column from Grid A to Grid 1 on 4th floor?	Grid-X, Grid-Y, FloorName, ColumnLabel
Inquiry: BeamSize	What’s the size of Girder G7 on foundation level? What’s the size of Beam B2 on 2nd floor?	What’s the size of the beam on Grid A from Grid 1 to Grid 2 on 3rd floor?	FloorName, BeamLabel, Grid-Along, Grid-From, Grid-To

**Table 2:** Intents and their Utterances with Labeled Entities in the Training Set-215.

Intents	Utterances with Labeled Entities
Inquiry: FloorHeight	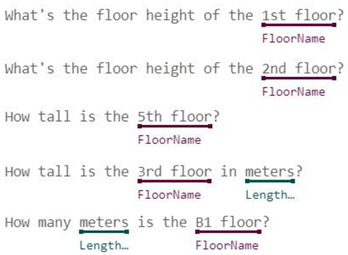
Inquiry: ColumnSize	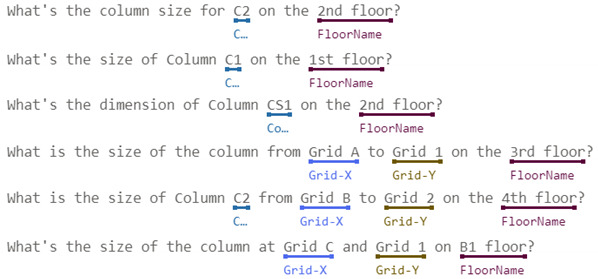
Inquiry: BeamSize	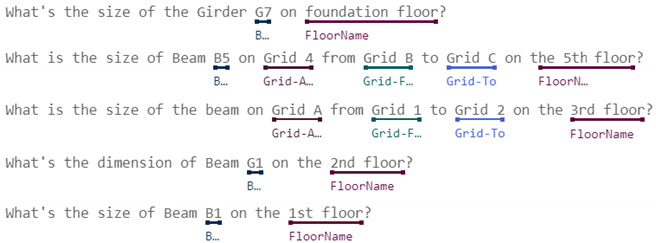

**Table 3:** Summary of utterances per intent in the dataset.

Intent	Training-215	Training-216	Testing
FloorHeight	5	8	7
ColumnSize	6	9	3
BeamSize	5	8	4
Trivial	5	5	3
Total	21	30	17

**Table 4:** Summary of total entities in the dataset.

Entity	Training-215	Training-216	Testing
CL	4	6	2
BL	4	7	4
FN	16	25	14
GX	4	4	1
GY	4	4	1
GA	2	2	1
GF	1	2	1
GT	1	2	1
LU	2	2	1
Total	38	54	26

**Table 5:** Newly-added utterances with labeled entities for the training dataset-216.

Intents	Utterances with Labeled Entities
Inquiry: FloorHeight	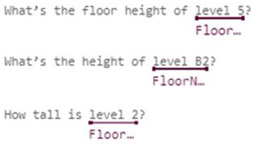
Inquiry: ColumnSize	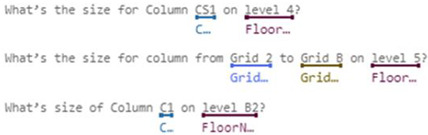
Inquiry: BeamSize	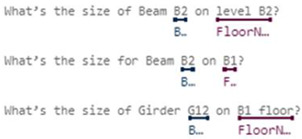

**Table 6:** Comparison of prediction performances between chatbot JULIE and ChatGPT.

Intents	Test No	Labeled Utterances	Entities in Top Intent Predicted by Chatbot JULIE	Entities Predicted by ChatGPT
Inquiry: FloorHeight		Named Method:		
	1-1	How tall is the 2nd floor?	“2nd floor” (floor name)	“tall” (object of inquiry, which is being used here as a synonym for “height”)
				“2nd floor” (specific floor)
	1-2	What’s the height of level 3?	“level 3” (floor name)	“height” (object of inquiry)
				“level 3” (specific level)
	1-3	What’s the floor height for B1?	“nil” (floor name)	“floor height” (object of inquiry)
				“B1” (specific floor)
Inquiry: ColumnSize		Named Method:		
	2-1	What’s size of Column C1 on 1st floor?	“C1” (column label) “1st floor” (floor name)	“size” (object of inquiry)
				“Column C1” (specific column)
				“1st floor” (specific floor)
	2-2	What’s the dimension for Column C3 on level 2?	“C3” (column label) “level 2” (floor name)	“dimension” (object of inquiry)
				“Column C3” (specific column)
				“level 2” (specific level)
		Grid Method:		
	2-3	What’s the size for column from Grid A to Grid 1 on 4th floor?	“Grid A” (grid-x) “Grid 1” (grid-y) “4th floor” (floor name)	“size” (object of inquiry)
				“column” (the element type)
				“Grid A” and “Grid 1” (the specific start and end grid references for the column)
				“4th floor” (specific floor)
Inquiry: BeamSize		Named Method:		
	3-1	What’s the size of Girder G7 on foundation level?	“G7” (beam label) “foundation level” (floor name)	“size” (object of inquiry)
				“Girder G7” (specific girder)
				“foundation level” (specific level)
	3-2	What’s the size of Beam B2 on 2nd floor?	“B2” (beam label) “2nd floor” (floor name)	“size” (object of inquiry)
				“Beam B2” (specific beam
				“2nd floor” (specific floor)
		Grid Method:		
	3-3	What’s the size of the beam on Grid A from Grid 1 to Grid 2 on 3rd floor?	“Grid A” (grid-along) “Grid 1” (grid-from) “Grid 2” (grid-to) “3rd floor” (floor name)	“size” (object of inquiry)
				“beam” (the element type)
				“Grid A” (specific grid)
				“Grid 1” and “Grid 2” (the specific start and end grid references for the beam)
				“3rd floor” (specific floor)

## Data Availability

Publicly available datasets were analyzed in this study. This data can be found here: https://github.com/DrWillTWN/JULIE (accessed on 27 February 2023).
